# Distinct functional roles for the M4 α-helix from each homologous subunit in the heteropentameric ligand-gated ion channel nAChR

**DOI:** 10.1016/j.jbc.2022.102104

**Published:** 2022-06-07

**Authors:** Mackenzie J. Thompson, Jaimee A. Domville, Claire H. Edrington, Angelica Venes, Patrick M. Giguère, John E. Baenziger

**Affiliations:** Department of Biochemistry, Microbiology, and Immunology, University of Ottawa, Ottawa, Ontario, Canada

**Keywords:** nAChR, pentameric ligand-gated ion channels, lipid–protein interactions, lipid sensing, M4, channel gating, α-BTX, α-bungarotoxin, ACh, acetylcholine, CMS, congenital myasthenic syndrome, cRNA, circular RNA, ECD, extracellular domain, ELIC, *Erwinia* ligand-gated ion channel, GLIC, *Gloebacter* ligand-gated ion channel, HEK293T, human embryonic kidney 293T cell line, nAChR, nicotinic acetylcholine receptor, pLGIC, pentameric ligand-gated ion channel, PRB, phosphate ringer buffer, TEVC, two-electrode voltage clamp, TMD, transmembrane domain

## Abstract

The outermost lipid-exposed α-helix (M4) in each of the homologous α, β, δ, and γ/ε subunits of the muscle nicotinic acetylcholine receptor (nAChR) has previously been proposed to act as a lipid sensor. However, the mechanism by which this sensor would function is not clear. To explore how the M4 α-helix from each subunit in human adult muscle nAChR influences function, and thus explore its putative role in lipid sensing, we functionally characterized alanine mutations at every residue in αM4, βM4, δM4, and εM4, along with both alanine and deletion mutations in the post-M4 region of each subunit. Although no critical interactions involving residues on M4 or in post-M4 were identified, we found that numerous mutations at the M4–M1/M3 interface altered the agonist-induced response. In addition, homologous mutations in M4 in different subunits were found to have different effects on channel function. The functional effects of multiple mutations either along M4 in one subunit or at homologous positions of M4 in different subunits were also found to be additive. Finally, when characterized in both *Xenopus* oocytes and human embryonic kidney 293T cells, select αM4 mutations displayed cell-specific phenotypes, possibly because of the different membrane lipid environments. Collectively, our data suggest different functional roles for the M4 α-helix in each heteromeric nAChR subunit and predict that lipid sensing involving M4 occurs primarily through the cumulative interactions at the M4–M1/M3 interface, as opposed to the alteration of specific interactions that are critical to channel function.

Although the functional sensitivity of the muscle-type (α_2_βγδ) nicotinic acetylcholine receptor (nAChR) from *Torpedo* to lipids has been extensively characterized (1, 2, 3), the mechanisms by which lipids influence function remain poorly understood. It is known that lipids alter function predominantly *via* a conformational selection mechanism whereby some membranes preferentially stabilize the activatable resting state, whereas others preferentially stabilize nonactivatable desensitized or uncoupled states (4, 5, 6, 7). Several observations also suggest that the M4 α-helix from each of the five subunits plays a central role in lipid sensing (8). M4 is located at the periphery of the transmembrane domain (TMD) of each subunit, where it forms extensive contacts with the lipid bilayer (Fig. 1). Numerous mutations in M4 influence channel function, including an αC418W potentiating mutation that leads to a congenital myasthenic syndrome (CMS) (9, 10, 11, 12, 13). Lipids are also observed bound to the interfaces between M4 and the adjacent M1 and M3 α-helices in the *Torpedo* nAChR and in other pentameric ligand-gated ion channels (pLGICs), although the functional roles of these bound lipids remain to be defined (3, 14, 15, 16, 17).

**Figure 1:**
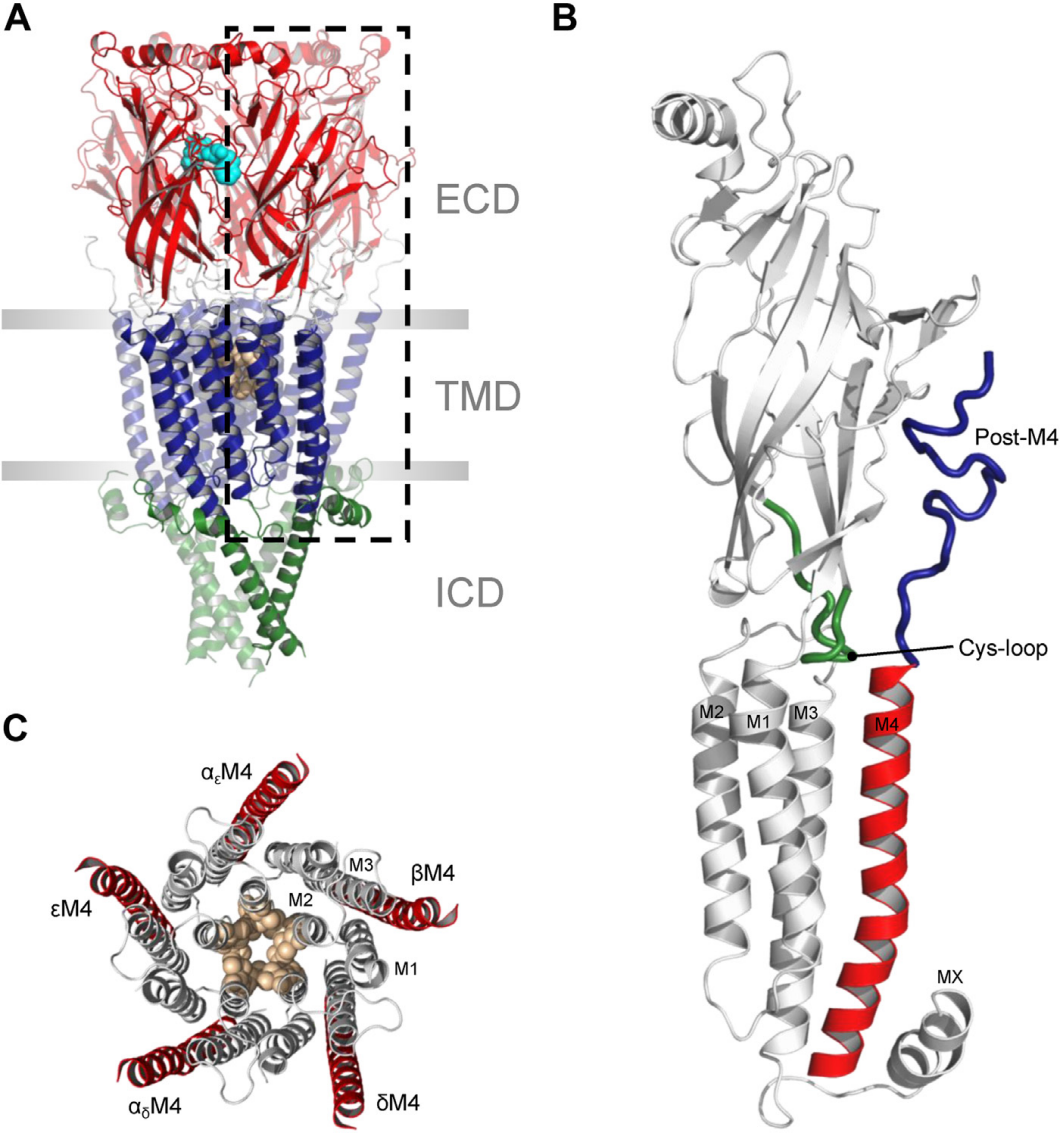
**The M4 lipid sensors from each subunit of the nAChR are the most lipid-exposed TMD α-helices.** Homology model of the human adult muscle nAChR based on the 2.7 Å resolution *Torpedo* nAChR structure (Protein Data Bank: 6UWZ). *A*, side view of the full model colored by domain with agonist-binding site residues (αTrp149) and channel gate residues (9′ and 13′) is shown as *spheres* colored *cyan* and *tan*, respectively. *B*, zoomed in view of a single subunit with the MA α-helix removed for clarity. The M4 α-helix, post-M4, and the Cys-loop are shown in *red*, *blue*, and *green*, respectively. *C*, top–down view of the TMD with M4 helices from each subunit colored *red*. nAChR, nicotinic acetylcholine receptor; TMD, transmembrane domain.

One plausible mechanism by which lipids influence nAChR function is by modulating interactions between M4 and the remainder of the TMD. More specifically, lipid-induced changes in the position of M4 relative to M1 and M3 could alter interhelical packing of the entire TMD in a manner that directly influences channel gating or desensitization, as was recently suggested for lipid binding to the M4–M1 interface of the prokaryotic pLGIC, *Erwinia* ligand-gated ion channel (ELIC) (18). Altered M4–M1/M3 interactions could also reposition the M4 C terminus (post-M4) to interact with structures in the extracellular domain (ECD) to alter the physical coupling between the agonist-binding ECD and channel-gating TMD (Fig. 1*B*). The latter hypothesis is supported by the observation that post-M4 is critical to folding and function in some pLGICs (19, 20, 21, 22, 23, 24), albeit not in others (25, 26).

As a first step toward understanding the mechanisms by which the nAChR senses its lipid environment, we set out to characterize the functional role of the M4 α-helix from each subunit in a heteropentameric muscle-type nAChR. In a previous publication, we probed the functional role of M4 from the α subunit (αM4) of the human adult muscle nAChR (26). Here, we extend this study to include M4 from each of the remaining β (βM4), δ (δM4), and ε (εM4) subunits. Through mutagenesis and electrophysiological recordings, we identify interactions between M4 and M1/M3 in each subunit that influence channel function and that could thus participate in lipid sensing, although no critical functional interactions were identified. In addition, we show that the functional effects of point mutations along each M4 or at homologous positions in M4 from different subunits are additive so that multiple simultaneous mutations add together leading to substantial functional effects. Finally, we show that the functional consequences of some M4 mutations are dependent upon the cellular context. Our data predict that lipid sensing in the muscle nAChR *via* M4 is governed by cumulative changes in multiple interactions at the M4–M1/M3 interface that add up to substantive functional effects, as opposed to the alteration of specific interactions that play a critical role in channel function.

## Results

### Alanine scan of αM4, βM4, δM4, and εM4

The M4 α-helix from each of the four nAChR subunits is composed predominantly of aliphatic residues interspersed with neutral hydrogen bonding, charged and aromatic residues that could each form interactions with side chains on M1/M3 or with lipids that are essential to channel function and that could thus play a role in lipid sensing. To identify functionally important interactions, we generated an alanine mutation of each residue on M4 from the α, β, δ, and ε subunits. We were generous in our definition of M4 and included several residues in flanking regions, including many in post-M4. We examined the functional consequences by expressing each M4-mutated subunit along with nonmutated subunits in *Xenopus* oocytes. The concentration response of each to acetylcholine (ACh) was measured using two-electrode voltage clamp (TEVC) electrophysiology.

**Figure 2:**
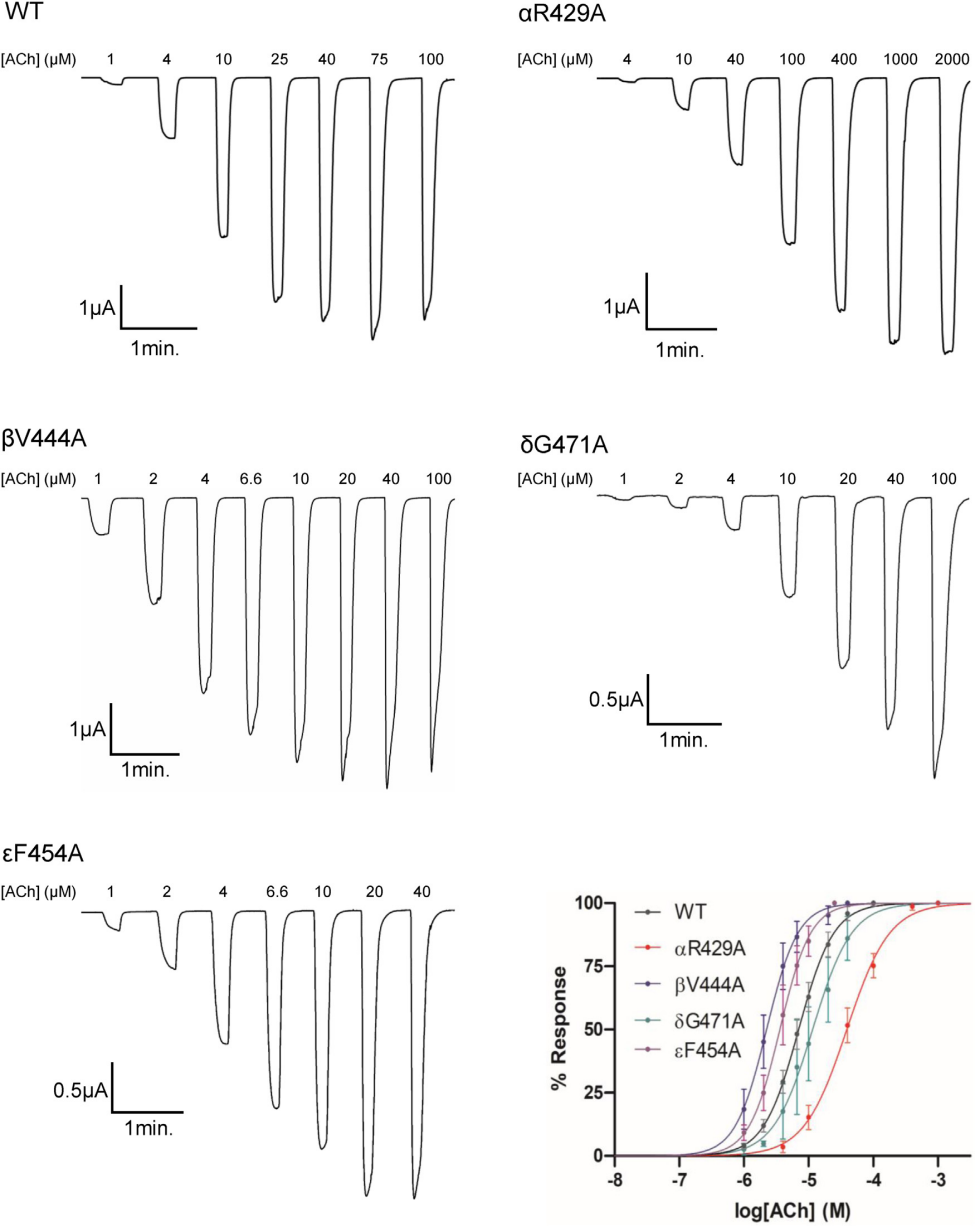
**Functional effects of alanine mutations to residues within each M4 α-helix of the nAChR.** Representative whole-cell two-electrode voltage clamp traces are shown for WT and the largest function-altering Ala mutants in the M4 α-helices of each subunit. Normalized concentration response curves for the selected mutants are shown in the *bottom right*. nAChR, nicotinic acetylcholine receptor.

Of the 155 generated alanine mutants (36 in α, 40 in β, 37 in δ, and 42 in ε), all but one (εM430A) functionally expressed, with each of the functional mutants leading to robust inward currents whose peak amplitudes increase in an ACh concentration–dependent manner (Fig. 2). Derived EC_50_/pEC_50_ values for those mutations that led to statistically significant changes in function are summarized in Table 1, with the EC_50_/pEC_50_ values for all mutations presented in Tables S1–S4. Note that each EC_50_/pEC_50_ value reflects a weighted ensemble of all the rate constants associated with both agonist binding/dissociation and channel opening/closing, although the measured values can be influenced by the rates of desensitization. We assume that the changes in the measured EC_50_/pEC_50_ values reflect primarily changes in the channel opening/closing rate constants as (1) the studied mutations are distant from the agonist-binding site and thus unlikely to directly alter agonist binding/dissociation (11, 27) and (2) although only minor changes in the rates of desensitization are observed (Fig. 2), the reported changes in EC_50_, or lack thereof, are not correlated with altered desensitization rates. A left shift in the concentration response leading to a decrease in EC_50_ reflects a gain of function, whereas a right shift leading to an increase in EC_50_ reflects a loss of function.

**Table 1:** Alanine mutations in M4 of each subunit that led to statistically significant changes in pEC_50_

Mutant	Dose response^a^^,^^b^				Fold change
	EC_50_ (μM)	pEC_50_ (M)	Hill slope	n	$\left[\frac{{\text{EC}}_{50}\left(\text{Mut}\right)}{{\text{EC}}_{50}\left(\text{WT}\right)}\right]$
WT	7.61	5.12 ± 0.07	1.70 ± 0.47	50	—
α subunit					
αL433A	10.3	4.99 ± 0.04	1.93 ± 0.14	12	1.35
αR429A	40.0	4.41 ± 0.11	1.09 ± 0.12	10	5.25
αF426A	2.02	5.71 ± 0.14	1.80 ± 0.76	11	0.27
αV425A	4.30	5.37 ± 0.05	1.89 ± 0.28	10	0.57
αL423A	5.80	5.24 ± 0.07	2.54 ± 0.66	10	0.76
αT422A	31.2	4.52 ± 0.13	1.40 ± 0.12	10	4.10
αG421A	10.0	5.00 ± 0.05	1.96 ± 0.26	9	1.32
αI420A	5.97	5.23 ± 0.07	2.71 ± 0.33	10	0.78
αC418A	10.6	4.99 ± 0.13	1.82 ± 0.33	9	1.40
αM415A	12.5	4.92 ± 0.13	1.61 ± 0.27	10	1.65
αF414A	4.47	5.36 ± 0.09	1.76 ± 0.34	10	0.59
αL411A	9.86	5.02 ± 0.10	2.35 ± 0.40	13	1.30
αL410A	5.21	5.27 ± 0.06	2.06 ± 0.59	21	0.68
αH408A	10.1	5.00 ± 0.05	1.80 ± 0.27	10	1.33
αD407A	4.97	5.31 ± 0.04	3.12 ± 0.78	11	0.65
αY401A	9.86	5.01 ± 0.03	1.75 ± 0.48	11	1.30
αK400A	12.9	4.90 ± 0.09	1.89 ± 0.63	15	1.70
β subunit					
βP476A	4.98	5.33 ± 0.20	1.49 ± 0.34	13	0.65
βD475A	4.15	5.39 ± 0.11	1.27 ± 0.31	8	0.54
βH470A	13.0	4.90 ± 0.11	1.24 ± 0.19	13	1.71
βD466A	5.08	5.31 ± 0.14	1.57 ± 0.48	11	0.67
βI463A	3.67	5.44 ± 0.16^b^	1.78 ± 0.11	8	0.48
βG459A	14.4	4.90 ± 0.28	1.12 ± 0.30	10	1.89
βS457A	13.2	4.93 ± 0.22	1.10 ± 0.24	11	1.74
βI453A	4.57	5.36 ± 0.12	1.46 ± 0.24	8	0.60
βW450A	12.7	4.94 ± 0.19	1.27 ± 0.20	14	1.66
βL449A	15.9	4.82 ± 0.15	1.45 ± 0.19	8	2.09
βF448A	4.36	5.38 ± 0.15	1.63 ± 0.48	8	0.57
βV444A	2.22	5.66 ± 0.07	1.89 ± 0.30	11	0.29
δ subunit					
δP478A	4.72	5.34 ± 0.11^b^	1.43 ± 0.56	7	0.62
δP477A	11.7	4.94 ± 0.09^b^	1.26 ± 0.14	8	1.51
δG471A	14.1	4.86 ± 0.11^b^	1.57 ± 0.25	4	1.85
δL469A	5.13	5.30 ± 0.09^b^	1.71 ± 0.38	9	0.67
δI467A	4.63	5.34 ± 0.07^b^	1.91 ± 0.79	7	0.61
δW466A	5.49	5.28 ± 0.14^b^	1.57 ± 0.27	8	0.72
δG463A	4.88	5.32 ± 0.10^b^	1.71 ± 0.38	8	0.64
δP458A	4.08	5.40 ± 0.09^b^	2.06 ± 0.75	7	0.54
δV456A	5.06	5.33 ± 0.19^b^	1.58 ± 0.41	8	0.66
δC452A	4.86	5.32 ± 0.07^b^	1.56 ± 0.16	11	0.64
δR450A	5.15	5.29 ± 0.04^b^	1.72 ± 0.17	7	0.68
δD449A	5.03	5.34 ± 0.20^b^	2.00 ± 0.19	8	0.66
δV444A	4.35	5.36 ± 0.03^b^	2.02 ± 0.17	8	0.57
ε subunit					
εI471A	15.5	4.81 ± 0.07^b^	1.61 ± 0.24	10	2.04
εC470A	13.4	4.88 ± 0.05^b^	1.54 ± 0.08	12	1.76
εP463A	12.1	4.93 ± 0.08^b^	1.77 ± 0.10	9	1.59
εY458A	5.10	5.30 ± 0.07^b^	1.43 ± 0.20	8	0.67
εF454A	3.90	5.42 ± 0.10^b^	1.80 ± 0.25	8	0.51
εI453A	4.50	5.35 ± 0.06^b^	1.83 ± 0.27	8	0.59
εG449A	5.62	5.26 ± 0.12^b^	1.54 ± 0.52	9	0.74
εC438A	10.1	5.00 ± 0.06^b^	1.67 ± 0.20	9	1.33
εN436A	12.9	4.90 ± 0.09^b^	1.76 ± 0.22	11	1.70
εG431A	10.6	4.99 ± 0.11^b^	1.60 ± 0.17	8	1.40
εM430A	No current^c^	-	-	8	-
εV428A	5.62	5.25 ± 0.06^b^	1.56 ± 0.19	8	0.74
εW427A	5.85	5.25 ± 0.14^b^	1.37 ± 0.39	9	0.77

a Measurements performed 1 to 4 days after cRNA injection (V_hold_ ranging from −20 to −80 mV). Error values are represented as standard deviation.

b *p* < 0.001 relative to WT *via* one-way ANOVA followed by Dunnet’s post hoc test.

c No significant current observed up to 4 days after cRNA injection.

As was observed previously with alanine substitutions in αM4 (26), alanine substitutions in βM4, δM4, and εM4 led to a mix of gain-of-function and loss-of-function phenotypes, with most of the function-altering mutations located along the M4–M1/M3 interface (Fig. S1). The proportion of mutations leading to statistically significant changes in function is slightly lower in β, δ, or ε than in α, which is present twice per pentamer (17 of 36 in α [47%]; 12 of 40 in β [30%]; 13 of 37 in δ [35%]; and 12 of 42 [29%] in ε). Furthermore, only four of the 119 mutations in β, δ, and ε combined led to more than a two-fold change in function (βV444A, βL449A, βI463A, and εI471A) with the largest being a 3.4-fold gain of function with βV444A. In contrast, three of 36 mutants do so in the α subunit, with these three mutants leading to larger 4.1-fold, 3.8-fold, and 5.3-fold changes in function (αT422A, αF426A, and αR429A, respectively). The detected changes in EC_50_ values show that there are interactions at both the M4–M1/M3 and M4–lipid interface that influence channel function. On the other hand, the absence of dramatic changes in the EC_50_ values (except for εM430A, see later) suggests that there are no specific interactions at either interface that are critical for channel gating.

**Figure 3:**
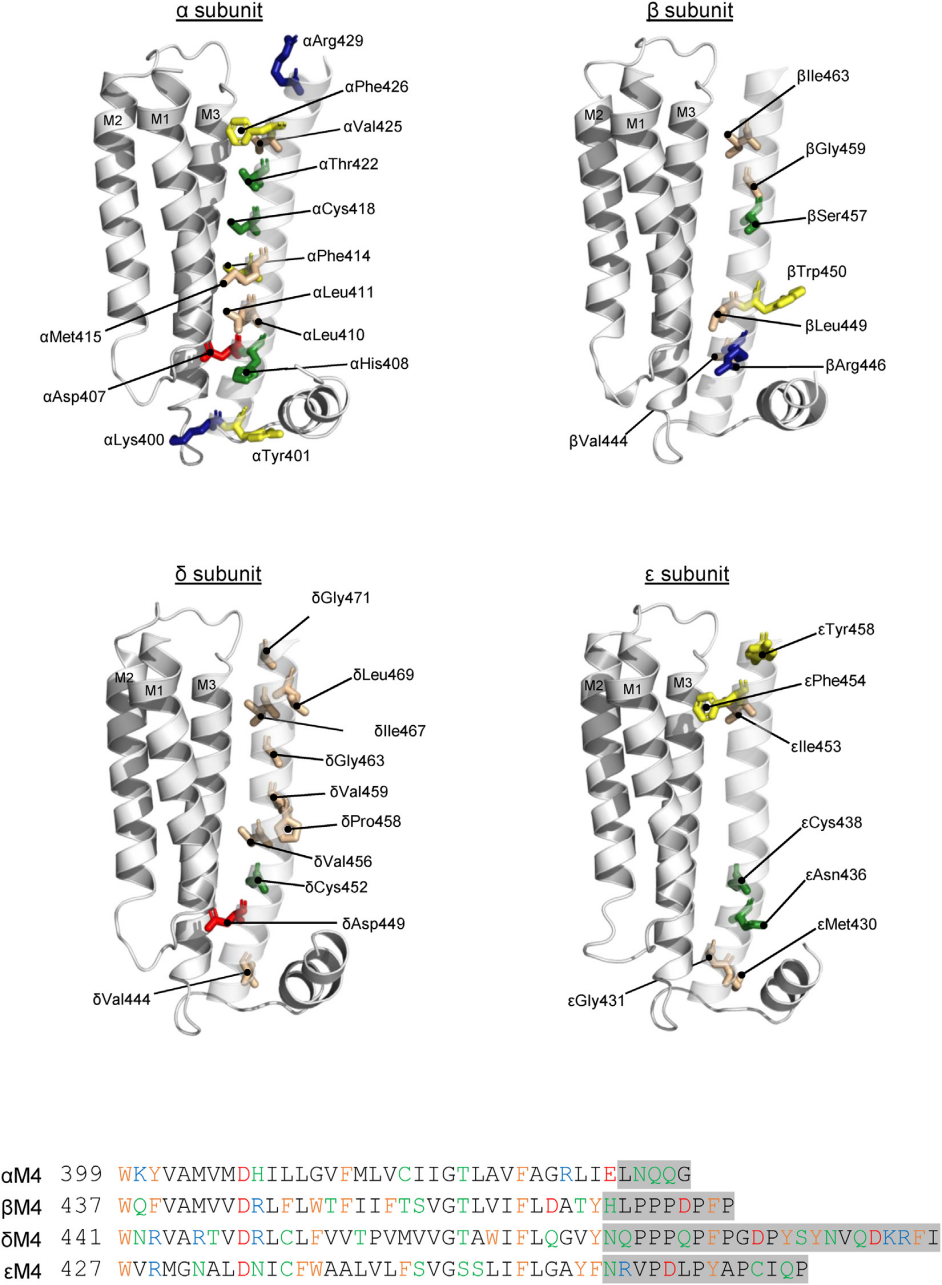
**Position of residues that cause significant changes in function when mutated to Ala.** Zoomed in views of each subunit’s TMD with residues from M4 that significantly altered the EC_50_ when mutated to Ala shown as *sticks* and colored according to residue type: aliphatic, *tan*; aromatic, *yellow*; polar/hydrogen bonding, *green*; negative, *red*; and positive, *blue*. A sequence alignment of M4 α-helices from each subunits of the human adult nAChR is shown at the *bottom* with residues colored according to residue type (aliphatic, *black*; aromatic, *yellow*; polar/hydrogen bonding, *green*; negative, *red*; and positive, *blue*) with post-M4 highlighted in *gray*. nAChR, nicotinic acetylcholine receptor; TMD, transmembrane domain.

The data exhibit several intriguing trends that allow us to glean some insight into the functional roles played by the M4 α-helix from each of the different subunits:

First, of the four alanine mutations in β/δ/ε that altered function by more than twofold, three of these are in βM4 (βV444A, βL449A, and βI463A) (Fig. 3). In contrast, although δM4 has a higher proportion of statistically significant function altering alanine mutants than βM4, none produced more than a twofold change of function. Furthermore, alanine mutations in εM4 led to relatively few statistically significant changes in function, although εI471A, which is in post-M4, alters the EC_50_ approximately twofold. The relatively large changes in function observed with the three alanine mutations in βM4 suggest that specific regions along the βM4–βM1/βM3 interface are functionally important. This finding was unexpected given that β is a structural subunit that is not directly involved in agonist binding. In addition, the four TMD α-helices in the β subunit undergo the lowest amplitude motions upon agonist binding (16, 17).

Second, none of the alanine mutations of residues in β, δ, and ε that align with those residues in αM4, whose mutation to alanine led to relatively large changes in function, have substantial effects on the measured EC_50_ values. Specifically, αT422A, αF426A, and αR429A led to 4.1-fold, 3.8-fold, and 5.3-fold changes in the recorded EC_50_ values, as noted previously. The equivalent residues in the other three subunits are βT460, βF464, and βA467; δT464, δF468, and δG471; and εS450, εF454, and εA457. Of the alanine mutations generated for these equivalent residues, only εF454A and δG471A led to statistically significant changes in the EC_50_ values, although the effects on function in both cases are less than twofold. These data show that identical changes in the structure of the M4 α-helix from different subunits lead to different effects on function. The M4 α-helix from the α, β, δ, and ε subunits thus each plays a subtly different functional role.

Third, εM430A is the only mutant that did not functionally express (Fig. 3). εMet430 extends toward εMX into a hydrophobic pocket formed by residues on εM3, εM4, and εMX. εMX is implicated in the assembly/cell surface trafficking of the muscle nAChR, with mutations in εMX reducing cell surface expression leading to CMS (28). Residues in M4 that project toward MX may play a particularly important role in nAChR expression.

Finally, we were surprised to see that the εC470A mutant led to robust ACh-induced currents that are comparable in magnitude to those observed with the WT nAChR. In contrast, εC470A, εC470S, and a deletion mutation at εC470 each inhibits cell surface expression of the nAChR in human embryonic kidney 293T (HEK293T) cells, with low expression of the latter in humans leading to CMS (29). It has been suggested that the sulfhydryl side chain of εCys470 is critical for folding and expression. Our data show that the side chain of εC470 is not intrinsically required for folding. It appears that the lipid environment of an oocyte supports folding of the εC470A mutant, whereas the lipid environments of HEK293T cells and muscle cells do not (see later).

### Role of post-M4 in channel function

Post-M4 is required for optimal expression/function in some pLGICs but not in others (19, 20, 21, 22, 23, 24). In our alanine scans, we observed that only nine of 51 mutations in post-M4 led to statistically significant changes in function (αL433A, βH470A, βD475A, βP476A, δP477A, δP478A, εP463A, εC470A, and εI471A), but none of these altered function by more than approximately twofold.

**Figure 4:**
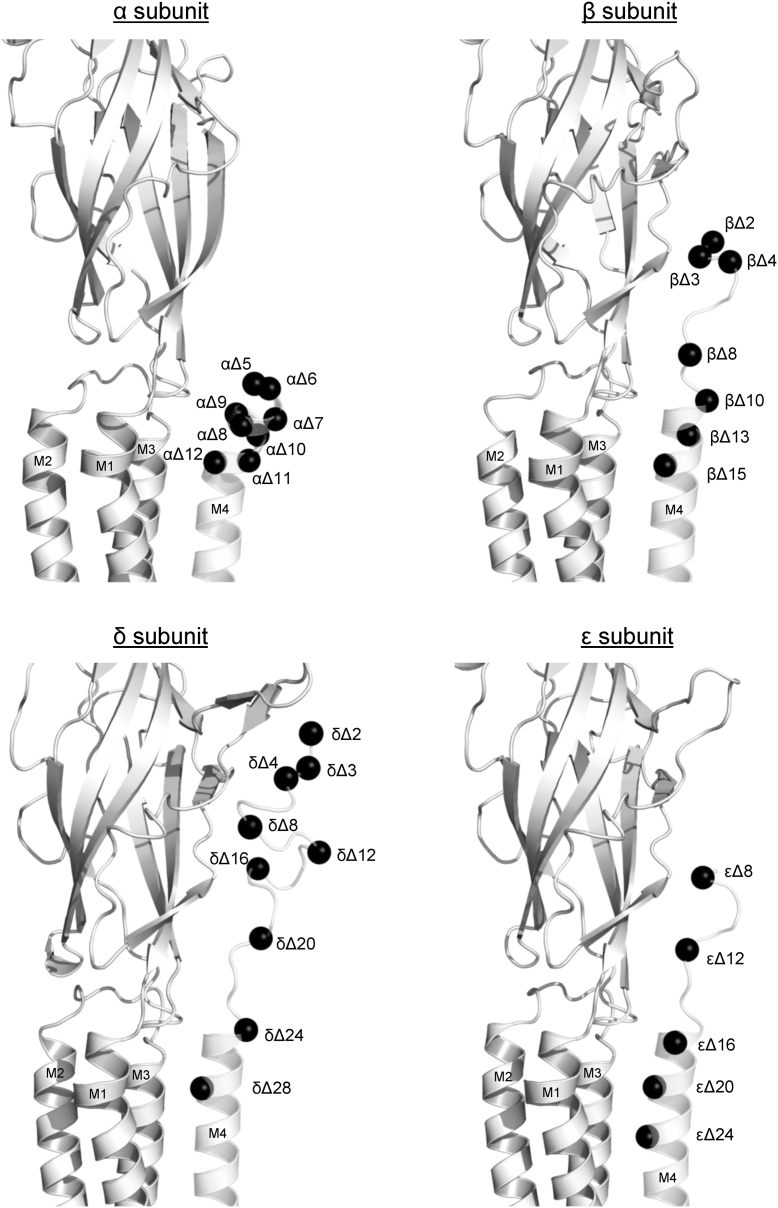
**Location of C-terminal deletions in each subunit.** Side views of each subunit are shown with M4 helices and post-M4 semitransparent. *Black spheres* denote α-carbons for each deletion mutation.

Although the subtle effects of the single alanine mutants imply that interactions between post-M4 and the remainder of the nAChR are not critical for folding/function, we explored this possibility further by generating a series of C-terminal deletions in each subunit. In the α subunit, deletion of up to nine residues (αΔ9) led to only a twofold or less loss of function, with the deletion of additional residues extending into the M4 α-helix (αΔ12) eventually leading to a loss of functional expression (26) (Fig. 4 and Table 2). Similarly, deleting up to eight residues in βM4 and εM4, or 12 residues in δM4, had little to no effect, with further deletions of up to 13 residues in βM4 and 24 residues in either δM4 or εM4 leading to subtle loss of function (β and δ) or gain of function (ε). Surprisingly, the 24-residue deletion in δ restored WT activity, whereas the 15- and 24-residue deletions in β and ε, respectively, led to gain of function (εΔ24 led to a relatively large 6.3-fold gain of function). These results show that the post-M4 region is not important in the folding or function of the adult muscle nAChR.

**Table 2:** Effects of M4 C-terminal deletions on nAChR function and expression

Dose response^a^				
Deletion(s)	EC_50_ (μM)	pEC_50_ (M)	Hill slope	n
α subunit				
WT: …LAVFAGRLIELNQQG	7.61	5.12 ± 0.07	1.70 ± 0.47	50
Δ1: …LAVFAGRLIELNQQ	6.86	5.17 ± 0.06	2.66 ± 0.67	9
Δ2: …LAVFAGRLIELNQ	6.37	5.20 ± 0.07	2.62 ± 0.54	9
Δ3: …LAVFAGRLIELN	7.14	5.15 ± 0.07	2.36 ± 0.38	8
Δ4: …LAVFAGRLIEL	8.49	5.08 ± 0.07	2.13 ± 0.44	8
Δ5: …LAVFAGRLIE	11.8	4.93 ± 0.04^b^	1.65 ± 0.33	10
Δ6: …LAVFAGRLI	12.3	4.91 ± 0.04^b^	1.59 ± 0.25	10
Δ7: …LAVFAGRL	12.7	4.90 ± 0.06^b^	1.54 ± 0.15	10
Δ8: …LAVFAGR	14.7	4.84 ± 0.07^b^	1.46 ± 0.29	10
Δ9: …LAVFAG	14.9	4.83 ± 0.08^b^	1.77 ± 0.35	10
Δ10: …LAVFA	21.4	4.68 ± 0.08^b^	1.35 ± 0.12	10
Δ11: …LAVF	23.0	4.65 ± 0.09^b^	1.69 ± 0.36	10
Δ12: …LAV	No current^c^	-	-	8
β subunit				
WT: …LVIFLDATYHLPPPDPFP	7.61	5.12 ± 0.07	1.70 ± 0.47	50
Δ1: …LVIFLDATYHLPPPDPF	9.77	5.02 ± 0.09	1.59 ± 0.08	13
Δ2: …LVIFLDATYHLPPPDP	9.65	5.02 ± 0.05	1.61 ± 0.11	11
Δ3: …LVIFLDATYHLPPPD	9.04	5.05 ± 0.08	1.46 ± 0.19	12
Δ4: …LVIFLDATYHLPPP	7.00	5.13 ± 0.15	1.75 ± 0.13	10
Δ8: …LVIFLDATYH	8.36	5.10 ± 0.16	1.79 ± 0.20	7
Δ10: …LVIFLDAT	11.6	4.94 ± 0.08^b^	1.45 ± 0.21	9
Δ13: …LVIFL	15.0	4.84 ± 0.14^b^	1.32 ± 0.30	11
Δ15: …LVI	4.25	5.33 ± 0.15^b^	1.87 ± 0.20	9
δ subunit				
WT: …WIFLQGVYNQPPPQPFPGDPYSYNVQDKRFI	7.61	5.12 ± 0.07	1.70 ± 0.47	50
Δ1: …WIFLQGVYNQPPPQPFPGDPYSYNVQDKRF	9.20	5.04 ± 0.07	1.46 ± 0.20	8
Δ2: …WIFLQGVYNQPPPQPFPGDPYSYNVQDKR	7.72	5.13 ± 0.13	1.71 ± 0.13	8
Δ3: …WIFLQGVYNQPPPQPFPGDPYSYNVQDK	5.95	5.24 ± 0.14	1.55 ± 0.10	8
Δ4: …WIFLQGVYNQPPPQPFPGDPYSYNVQD	8.11	5.10 ± 0.07	1.60 ± 0.21	8
Δ8: …WIFLQGVYNQPPPQPFPGDPYSY	8.88	5.06 ± 0.07	1.50 ± 0.33	9
Δ12: …WIFLQGVYNQPPPQPFPGD	9.90	5.01 ± 0.06	1.32 ± 0.18	8
Δ16: …WIFLQGVYNQPPPQP	14.2	4.85 ± 0.07^b^	1.68 ± 0.24	8
Δ20: …WIFLQGVYNQP	15.1	4.84 ± 0.12^b^	1.51 ± 0.20	8
Δ24: …WIFLQGV	7.59	5.12 ± 0.01	1.55 ± 0.13	3
Δ28: …WIF	No current^c^	—	—	8
ε subunit				
WT: …SVGSSLIFLGAYFNRVPDLPYAPCIQP	7.61	5.12 ± 0.07	1.70 ± 0.47	50
Δ1: …SVGSSLIFLGAYFNRVPDLPYAPCIQ	7.86	5.11 ± 0.05	1.62 ± 0.10	8
Δ2: …SVGSSLIFLGAYFNRVPDLPYAPCI	6.40	5.20 ± 0.05	1.72 ± 0.08	8
Δ3: …SVGSSLIFLGAYFNRVPDLPYAPC	8.44	5.08 ± 0.07	1.67 ± 0.11	9
Δ4: …SVGSSLIFLGAYFNRVPDLPYAP	9.26	5.04 ± 0.06	1.47 ± 0.17	8
Δ8: …SVGSSLIFLGAYFNRVPDL	6.20	5.22 ± 0.10	1.75 ± 0.25	8
Δ13: …SVGSSLIFLGAYFN	3.47	5.46 ± 0.05^b^	1.87 ± 0.24	8
Δ16: …SVGSSLIFLGA	4.22	5.38 ± 0.06^b^	1.87 ± 0.22	8
Δ18: …SVGSSLIFL	1.89	5.74 ± 0.13^b^	1.99 ± 0.16	9
Δ20: …SVGSSLI	1.21	5.92 ± 0.09^b^	1.87 ± 0.37	4
Δ24: …SVG	No current^c^	—	—	8

a Measurements performed 1 to 4 days after cRNA injection (V_hold_ ranging from −20 to −80 mV). Error values are represented as standard deviation.

b *p* < 0.001 relative to WT *via* one-way ANOVA followed by Dunnet’s post hoc test.

c No significant current observed up to 4 days after cRNA injection.

### Aromatic residues at the M4–M1/M3 interface

Aromatic residues play a critical role at the M4–M1/M3 interface in many pLGICs. Some pLGICs, such as the 5-HT_3_AR, the α1 GlyR, the α7 nAChR, the ρ1 GABA_A_R, and the prokaryote *Gloebacter* ligand-gated ion channel (GLIC), exhibit an extensive network of interacting aromatic residues that drives M4–M1/M3 interactions to facilitate folding and possibly function (20, 23, 30, 31, 32, 33). In contrast, fewer aromatic residues at this interface in ELIC are thought to sterically prevent tight interactions between M4 and M1/M3, thus creating a more malleable M4–M1/M3 interface that is potentially more sensitive to modulation by factors, such as the surrounding lipid environment (34). As in ELIC, the muscle nAChR exhibits relatively few aromatic residues likely leading to a malleable M4–M1/M3 interface that might underlie its exquisite lipid sensitivity (31).

**Figure 5:**
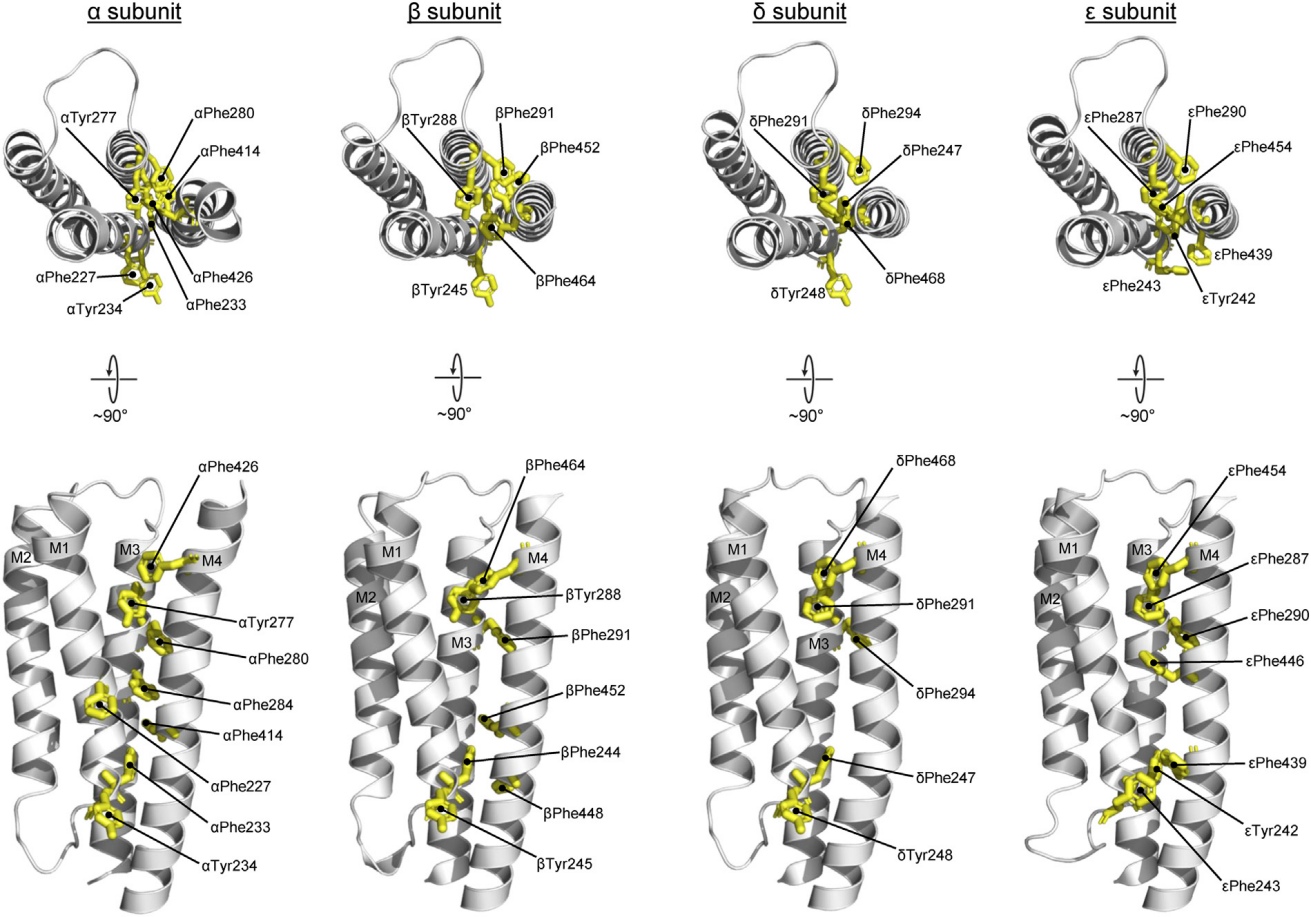
**Aromatic residues along the M4–M1/M3 interface in each subunit.** Top–down (*top*) and side (*bottom*) views of each subunit’s TMD with aromatic residues at the M4–M1/M3 interface shown as *yellow sticks*. TMD, transmembrane domain.

We mutated every aromatic residue at this interface in each subunit of the nAChR to alanine and tested the effects of each on channel function (Fig. 5 and Table 3). In general, we found that aromatic to alanine substitutions in the α, β, δ, and ε subunits led to either no effect or subtle gains in function. These data suggest that bulky aromatic side chains sterically prevent optimal M4–M1/M3 interactions, with the reduction in size possibly promoting tighter interactions to enhance channel function.

**Table 3:** Effects of mutating aromatic residues at the M4–M1/M3 interface on nAChR function

Dose response^a^					
Mutation	TMD α-helix	EC_50_ (μM)	pEC_50_ (M)	Hill slope	n
WT		7.61	5.12 ± 0.07	1.70 ± 0.47	50
α subunit					
αF227A	M1	6.51	5.20 ± 0.09	1.43 ± 0.16	8
αF233A	M1	2.63	5.58 ± 0.05^b^	1.63 ± 0.20	9
αY234A	M1	No current^c^	—	—	8
αY277A	M3	11.2	4.96 ± 0.08^b^	1.78 ± 0.58	8
αF280A	M3	4.25	5.38 ± 0.11^b^	1.56 ± 0.29	8
αF284A	M3	4.80	5.32 ± 0.07^b^	1.33 ± 0.21	9
αF414A	M4	4.47	5.36 ± 0.09^b^	1.76 ± 0.34	10
αF426A	M4	2.02	5.71 ± 0.14^b^	1.80 ± 0.76	11
β subunit					
βF244A	M1	4.46	5.35 ± 0.14^b^	2.23 ± 0.77	9
βY245A	M1	7.44	5.13 ± 0.10	1.56 ± 0.16	7
βY288A	M3	5.43	5.27 ± 0.10^b^	1.77 ± 0.30	8
βF291A	M3	10.5	4.98 ± 0.09^b^	1.66 ± 0.35	9
βF448A	M4	4.36	5.38 ± 0.15^b^	1.63 ± 0.48	8
βF452A	M4	10.5	4.98 ± 0.07	1.69 ± 0.11	8
βF464A	M4	8.55	5.08 ± 0.11	1.38 ± 0.19	10
δ subunit					
δF247A	M1	11.2	4.95 ± 0.10^b^	1.45 ± 0.12	9
δY248A	M1	3.67	5.44 ± 0.12^b^	1.57 ± 0.22	9
δF291A	M3	6.18	5.21 ± 0.08	1.62 ± 0.16	9
δF294A	M3	3.86	5.41 ± 0.07^b^	1.72 ± 0.13	8
δF468A	M4	5.83	5.25 ± 0.10	1.60 ± 0.27	7
ε subunit					
εY242A	M1	8.12	5.09 ± 0.04	1.67 ± 0.11	10
εF243A	M1	8.02	5.10 ± 0.10	1.78 ± 0.19	9
εF287A	M3	12.7	4.90 ± 0.06^b^	1.51 ± 0.13	9
εF290A	M3	6.14	5.21 ± 0.12	2.06 ± 0.36	7
εF439A	M4	8.59	5.07 ± 0.07	1.81 ± 0.08	8
εF446A	M4	8.89	5.06 ± 0.07	1.51 ± 0.18	9
εF454A	M4	3.90	5.42 ± 0.10^b^	1.80 ± 0.25	8

a Measurements performed 1 to 4 days after cRNA injection (V_hold_ ranging from −20 to −80 mV). Error values are represented as standard deviation.

b *p* < 0.001 relative to WT *via* one-way ANOVA followed by Dunnet’s post hoc test.

c No significant current observed up to 4 days after cRNA injection.

### αC418W-induced potentiation of channel function

To compare further how similar changes in the structure of the M4 α-helix from different subunits influence channel function, we focused on a site where the introduction of a tryptophan in the α subunit, αC418W, potentiates channel function 16- to 25-fold leading to a slow channel CMS (13) (Table 4). Mutant cycles show that the αC418W-induced potentiation is driven primarily by a new interaction that forms between the introduced tryptophan, αTrp418, and an adjacent residue on αM1, αSer226, with this interaction likely stabilizing the open state (35). The importance of this interaction in αC418W-induced potentiation is demonstrated by the fact that αC418W only potentiates channel function 3.4-fold when the tryptophan is introduced onto the αS226A background.

**Table 4:** Interactions between the αC418W mutant and its equivalents and adjacent residues from M1

Dose response^a^									Fold change
Background	WT				αC418W				$\left[\frac{{\text{EC}}_{50}\left(\text{WT}\right)}{{\text{EC}}_{50}\left(\text{mut}\right)}\right]$
	EC_50_ (μM)	pEC_50_ (M)	Hill slope	n	EC_50_ (μM)	pEC_50_ (M)	Hill slope	n	
WT	7.61	5.12 ± 0.07	1.70 ± 0.47	50	0.47	6.33 ± 0.13^b^	1.54 ± 0.23	50	16.2
αS226A	12.3	4.92 ± 0.11^b^	1.70 ± 0.47	8	3.66	5.45 ± 0.11^b^	1.26 ± 0.12	8	3.4
	WT				βT456W				
WT	7.61	5.12 ± 0.07	1.70 ± 0.47	50	4.50	5.36 ± 0.10^b^	1.73 ± 0.17	10	1.7
βT237A	6.28	5.22 ± 0.16	1.77 ± 0.10	8	4.26	5.38 ± 0.07^b^	1.59 ± 0.11	8	1.5
	WT				δM460W				
WT	7.61	5.12 ± 0.07	1.70 ± 0.47	50	12.1	4.93 ± 0.12^b^	1.41 ± 0.16	9	0.6
δS240A	No current^c^	—	—	8	5.89	5.23 ± 0.07	1.83 ± 0.17	4	—
	WT				εF446W				
WT	7.61	5.12 ± 0.07	1.70 ± 0.47	50	4.26	5.39 ± 0.14^b^	1.75 ± 0.13	9	1.8
εS235A	1.39	5.86 ± 0.06^b^	1.78 ± 0.43	4	0.38	6.43 ± 0.11^b^	1.74 ± 0.47	8	3.7

a Measurements performed 1 to 4 days after cRNA injection (V_hold_ ranging from −20 to −80 mV). Error values are represented as standard deviation.

b *p* < 0.001 relative to WT *via* one-way ANOVA followed by Dunnet’s post hoc test.

c No significant current observed up to 4 days after cRNA injection.

Given that β, δ, and ε each contains a homologous residue to αSer226 (βThr237, δSer240, and εSer235) on M1, we expected a similar degree of potentiation upon mutation of the αCys418 equivalent residue in each subunit (βThr456, δMet460, and εPhe446) to tryptophan. In contrast, tryptophan substitutions in β, δ, and ε, (βT456W, δM460W, and εF446W) led to only a 1.7-fold gain, a 1.6-fold loss, and a 1.8-fold gain of function, respectively, consistent with what is observed in the *Torpedo* nAChR (36, 37, 38). Furthermore, the βT237A mutation on M1 had no effect on the magnitude of the βT456W-induced response implying that the introduced tryptophan, βT456W, does not interact with βT237 to potentiate channel function. In the δ subunit, the δS240A mutant on M1 did not functionally express. In contrast, the εS235A mutation in M1 of the ε subunit enhances εF446W-induced potentiation suggesting that an interaction between F446W and εS235 is detrimental to εF446W-induced potentiation. These data illustrate how even analogous changes in the structure of M4 from different subunits can lead to different effects on channel function.

### M4 mutations in different subunits are additive

We previously observed with that the subtle functional effects of individual alanine mutations along αM4 are additive and thus can cumulatively lead to much larger changes in channel function. This implies that a reorientation of M4 could modulate many interactions at the M4–M1/M3 interface with functional effects of the individual alterations in structure adding up to a more substantial effect. Here, we tested whether mutations on different M4 α-helices are additive. Specifically, we focused on three positions where individual mutations in αM4 (αF426A, αV425A, and αC418W) lead to relatively large changes in function. We produced the equivalent mutations in the remaining β, δ, and ε subunits and then assessed the effect on function when all mutated subunits were expressed at the same time.

A phenylalanine at equivalent positions near the C terminus of M4 in all four subunits, αPhe426, βPhe464, δPhe468, and εPhe454, projects toward αM1 and αM3. The alanine mutation of each residue individually led to a 3.8-fold gain-, a 1.1-fold loss-, a 1.3-fold gain-, and a 2.0-fold gain-of-function, respectively. The simultaneous quadruple mutant, αF426A + βF464A + δF468A + εF454A, led to an 11.4-fold gain of function (Table 5), which is close to the 8.6-fold gain of function predicted if the functional effects of the mutations are independent and thus additive. Similarly, the adjacent αV426A, βI464A, δI468A, and εI454A mutants individually led to a 1.8-fold, a 2.1-fold, a 1.6-fold, and a 1.7-fold gain of function, respectively, with the quadruple αV426A + βI464A + δI468A + εI454A mutant leading to a 12.1-fold gain of function, again a value close to the 10.2-fold gain of function expected for independent additive mutations. Finally, the αC418W, βT456W, δM460W, and εF446W mutants noted previously led to a 16-fold gain-, a 1.7-fold gain-, a 1.6-fold loss-, and a 1.8-fold gain-of-function, respectively. The quadruple αC418W + βT456W + δM460W + εF446W mutant led to a 30.0-fold gain of function, virtually the same as that predicted (30.7-fold) for independent additive mutations.

**Table 5:** Mutations to aligned residues in each M4 α-helix have independent effects on function

Dose response^a^					Fold change	
Mutation(s)	EC_50_ (μM)	pEC_50_ (M)	Hill slope	n		
WT	7.61	5.12 ± 0.07^b^	1.70 ± 0.47	50	Observed	Predicted^c^
αF426A	2.02	5.71 ± 0.14^b^	1.80 ± 0.76	11	3.76	—
βF464A	8.55	5.08 ± 0.11	1.38 ± 0.19	10	0.89	—
δF468A	5.83	5.25 ± 0.10	1.60 ± 0.27	7	1.31	—
εF454A	3.90	5.42 ± 0.10^b^	1.80 ± 0.25	8	1.95	—
αF426A + βF464A + δF468A + εF454A	0.67	6.17 ± 0.17^b^	2.04 ± 0.15	8	11.4	8.54
αV425A	4.30	5.37 ± 0.05^b^	1.89 ± 0.28	10	1.77	—
βI463A	3.67	5.44 ± 0.16^b^	1.78 ± 0.11	8	2.07	—
δI467A	4.63	5.34 ± 0.07^b^	1.91 ± 0.79	7	1.65	—
εI453A	4.50	5.35 ± 0.06^b^	1.83 ± 0.27	8	1.69	—
αV425A + βI463A + δI467A + εI453A	0.63	6.20 ± 0.17^b^	2.11 ± 0.28	7	12.1	10.2
αC418W	0.47	6.33 ± 0.13^b^	1.54 ± 0.23	50	16.2	—
βT456W	4.50	5.36 ± 0.10^b^	1.73 ± 0.17	10	1.69	—
δM460W	12.2	4.93 ± 0.12^b^	1.41 ± 0.16	9	0.63	—
εF446W	4.26	5.39 ± 0.14^b^	1.75 ± 0.13	9	1.79	—
αC418W + βT456W + δM460W + εF446W	0.25	6.60 ± 0.08^b^	1.90 ± 0.58	9	30.0	30.7

a Measurements performed 1 to 4 days after cRNA injection (V_hold_ ranging from −20 to −80 mV). Error values are represented as standard deviation.

b *p* < 0.001 relative to WT *via* one-way ANOVA followed by Dunnet’s post hoc test.

c Predicted fold change if individual mutations affect function independently.

### M4 mutations have different effects on nAChR function in different membrane environments

Recent studies have shown that mutations in the M4 α-helix of the homopentameric 5-HT3_A_ receptor have different effects on function when the receptor is expressed in HEK293T cells *versus Xenopus* oocytes, with the different phenotypes attributed to the different lipid compositions of the plasma membranes (39). To determine if the functional effects of M4 mutations in the muscle nAChR are also dependent on their cellular context, we characterized six αM4 mutants in HEK293T cells using a membrane potential–sensitive fluorescent dye (Fig. S2). Although the measured EC_50_ values obtained using the fluorescent dye differ from those measured using TEVC electrophysiology in oocytes, we observed that two single αM4 Ala mutants, αF414A and αF426A, gave rise to similar fold changes in EC_50_ values relative to the WT nAChR in both heterologous expression systems (Table 6). In contrast, both αD407A and αR429A did not give rise to an agonist-induced response. [^125^I]-α-bungarotoxin (α-BTX; PerkinElmer) binding showed that while αR429A did not express, αD407A expressed well above background levels (Table 6). The αD407A mutant receptors that do reach the cell surface are thus unable to produce an agonist-induced response. Even though αD407A leads to a slight gain of function when expressed in *Xenopus* oocytes, the same αD407A mutation renders the nAChR inactive in HEK293T cells.

**Table 6:** Effects of M4 mutations on nAChR function and expression in HEK293T cells

Mutation(s)	Dose response^a^					[^125^I]-α-BTX^b^
	EC_50_ (μM)	pEC_50_ (M)	Hill slope	n	Fold change	CPM_mutant_/CPM_WT_
WT	0.30	6.54 ± 0.13	1.58 ± 0.32	9	—	1.00 ± 0.07^c^
αD407A	—	—d	—	3	—	0.23 ± 0.01^c^^,^^e^
αF414A	0.33	6.48 ± 0.06	1.41 ± 0.22	4	1.10	—
αF426A	0.09	7.04 ± 0.09^e^	1.12 ± 0.22	3	0.31	—
αR429A	—	—d	—	3	—	0.08 ± 0.04^e^
αF426A + F414A + D407A	—	—d	—	3	—	0.24 ± 0.08^c^^,^^e^
αR429A + T422A + L411A	2.31	5.64 ± 0.12^e^	1.87 ± 0.40	3	7.68	—
Untransfected	—	—d	—	9	—	0.08 ± 0.03^e^

a Measurements performed 2 days post-transfection. Error values are represented as standard deviation.

b Measurements performed 2 days post-transfection in triplicate. Error values are represented as standard deviation.

c *p* < 0.001 relative to untransfected cells *via* one-way ANOVA followed by Dunnet’s post hoc test.

d No agonist-induced response was observed.

e *p* < 0.001 relative to WT *via* one-way ANOVA followed by Dunnet’s post hoc test.

We also examined the functional effects of two triple M4 mutants. The first triple mutant, αL411A + αT422A + αR429A, led to a similar loss in function in both cell types (eightfold and sixfold loss of function in HEK293T cells *versus* oocytes, respectively). In contrast, the second triple mutant, αD407A + αF414A + αF426A, led to a complete loss of a response in HEK293T cells despite expressing at levels consistent with the αD407A mutant. Overall, the data show that the functional effects of select mutations within αM4 in the human muscle nAChR are different in HEK293T cells and oocytes.

## Discussion

The goal of this work was to probe how the structure of the M4 α-helix from each of the four distinct nAChR subunits influences channel function as a foundation for understanding the role played by each as a lipid sensor. In particular, we hoped to identify putative interactions involving residues on each M4 that are essential to channel function and that could be modulated by lipids to stabilize the nonactivatable uncoupled state that forms in phosphatidylcholine membranes lacking cholesterol and anionic lipids (5). To identify interactions that are essential to channel function, we generated alanine mutations of every M4 residue in each subunit. Surprisingly, all the generated mutants expressed robustly in frog oocytes except for one, εM430A, which extends toward a structure, εMX, that has been implicated in assembly/cell surface trafficking (28). Of those that expressed, 54 of 155 mutations led to statistically significant changes in the measured EC_50_ values and thus in channel function. Of these, only eight, however, led to shifts in EC_50_ values greater than approximately twofold, with αT422A and αR429A leading to 4.1-fold and 5.3-fold loss-of-function, respectively, and αF426A and βV444A leading to 3.8-fold and 3.4-fold gains-in-function, respectively. Although the detected changes in EC_50_ values confirm that interactions involving residues on M4 from each subunit influence channel function, there are likely no essential individual interactions that could be modulated by lipids to form the uncoupled state.

We also examined whether the post-M4 sequence in each subunit, which extends above the lipid bilayer, forms interactions with the ECD that are important to channel gating. We created a total of 51 Ala mutations in the post-M4 segments of the α, β, δ, and ε subunits, but all 51 of these mutants led to functional nAChRs with none altering the measured EC_50_ values by more than approximately twofold. Furthermore, deleting various regions or the entire post-M4 segment from any subunit (αΔ5, βΔ10, δΔ24, and εΔ16) had minimal detrimental effects on the measured EC_50_ values. In fact, some deletions, such as εΔ24, led to relatively large (6.3-fold) gains of function. These results suggest that there are no functionally essential interactions involving residues in post-M4 from any subunit.

The lack of essential interactions involving residues on M4 or post-M4 contrasts what has been observed in other pLGICs (23, 30, 32, 33, 40) and leads to a question as to how some lipid environments stabilize a nonactivatable uncoupled state. One possibility is that lipid-dependent uncoupling results from the cumulative effects of many changes in interactions involving residues on M4 that individually have only subtle impacts on channel function. This possibility is supported by two observations. First, the functional effects of multiple alanine substitutions on a single M4 α-helix are additive with simultaneous mutations leading to more pronounced effects on channel function, in some cases actually preventing functional expression altogether (26). Second, the functional effects of mutations of residues on the M4 α-helices from different subunits are additive with multiple simultaneous mutations leading to large cumulative effects. For example, simultaneous mutations of residues in each subunit equivalent to αV425A, αF426A, or αC418W led to 12.1-, 11.4-, and 30.4-fold changes in the recorded EC_50_ values, each close to the 10.2-, 8.6-, and 30.6-fold change in function predicted if the effect of each mutation is independent. Further work will be required to understand how cumulative changes to many subtle interactions involving M4 ultimately influence channel function.

On the other hand, it is intriguing to note that of the 173 alanine mutations characterized in this report, two led to nAChRs that did not functionally express in oocytes. One of the mutants, εM430A (εM4), likely impacts on nAChR assembly/cell surface trafficking. On the other hand, both εM430A and the other nonfunctional expressing mutant, αY234A (αM1), are located near the cytoplasmic surface of the bilayer close to newly identified phospholipid-binding sites on the *Torpedo* nAChR and cholesterol-binding sites on the α4β2 and α3β4 nAChRs (14, 16, 17, 41, 42). In fact, αY234 is thought to form part of a phospholipid-binding motif. The lack of functional expression of both these mutants may suggest that impaired lipid binding influences nAChR folding. Such lipid-binding sites could also play a role in lipid sensing. Further studies are currently aimed toward defining the roles of these lipid-binding sites in nAChR function.

Our mutational studies reveal additional features that impact on our understanding of potential mechanisms of lipid sensing *via* M4. First, our data reveal a common theme that a mutation in M4 from one subunit can have a different effect on function than the analogous mutation in a different subunit. For example, alanine substitutions of αR429, αF426, and αT422A lead to a 5.3-fold loss-, a 3.8-fold gain-, and a 4.1-fold loss of function, respectively. In contrast, alanine substitutions at equivalent sites in βM4 (βT460, βF464, and βA467), δM4 (δT464, δF468, and δG471), and εM4 (εS450, εF454, and εA457) have virtually no effect. Even more striking, while the CMS-causing mutation on αM4, αC418W, potentiates channel function 15- to 25-fold primarily through a stabilizing interaction with an adjacent serine residue, αSer226, on αM1, the analogous tryptophan substitutions in other subunits have little effect on function despite the presence of a homologous serine residue or threonine residue at the same position on M1 in each of the β (βThr237), δ (δerS240), and ε (εSer235) subunits. The lack of conservation of function despite a conserved structural motif suggests that the TMD α-helices from each subunit undergo different motions upon channel activation, thus leading to different poses of the M4 α-helix from different subunits relative to their adjacent M1 and M3 α-helices. In agreement, recent cryo-EM structures of the *Torpedo* nAChR solved in the presence and absence of agonist reveal subunit-specific tertiary deformations in each TMD (16, 17). These findings suggest that the same lipid-induced change in M4 structure in one subunit could have a strikingly different effect on channel function in another subunit.

Second, we found that alanine substitutions of bulky aromatic residues at the M4–M1/M3 interface typically led to subtle and more variable effects on nAChR function (11 of 27 significantly potentiates function) than in some pLGICs. For example, the glycine receptor and the prokaryotic homolog, GLIC, exhibit a complex network of interacting aromatic residues at this interface that is essential to folding and function. In these pLGICs, alanine substitutions of M4–M1/M3 interfacial aromatic residues invariably lead to losses of function, with multiple substitutions typically leading to a complete loss of functional expression (23, 30). Other pLGICs, such as the prokaryotic pLGIC ELIC, however, have relatively few aromatic residues. In the latter, aromatic to alanine substitutions invariably lead to gains in function suggesting that the bulky aromatic side chains sterically block the formation of M4–M1/M3 interactions that are optimal for channel function (31). Furthermore, the introduction of aromatic residues at the M4–M1/M3 interface in ELIC to mimic the complex aromatic network observed in GLIC not only enhanced ELIC function but renders ELIC less functionally sensitive to its membrane environment (34). While the trends observed with GLIC and ELIC are not adhered to strictly in all pLGICs (23, 30, 32, 33, 40), they have led to the suggestion that a more malleable M4–M1/M3 interface because of a lack of may lead to a more lipid-sensitive pLGIC. Our data show that as in ELIC, aromatic-to-alanine substitutions are well tolerated in the nAChR, consistent with a more malleable M4–M1/M3 interface that may contribute to a higher sensitivity to its surrounding lipid environment.

Finally, we characterized the effects of select αM4 mutations on nAChR function and expression in HEK293T cells to determine if these mutations have different effects when in membranes that differ in their lipid composition. Previous studies have shown that the effects of M4 mutations in the 5-HT_3A_R are different when expressed in HEK293T cells *versus* oocytes (39). Specifically, certain mutations that cause large shifts in EC_50_ or lead to nonfunctional receptors in HEK293T cells often have little to no influence on function in oocytes. In agreement, we find that mutations in αM4 that have little effect on nAChR function in oocytes, such as the αD407A and αR429A, cause a dramatic reduction in function in HEK293T cells. Similar trends have also been observed with other mutations, such as εC470A and βD445A, δD449A and εD435A, both here and in other studies (29, 43).

The observed difference in the functional effects of M4 mutations in HEK293T cells *versus* oocytes can be attributed to several factors, including different intracellular chaperones, proximal membrane proteins, or the lipid composition of the surrounding membrane. While speculative, we favor the latter hypothesis given that the mutations we have investigated here are within the lipid-exposed αM4 helix. In addition, previous studies have shown that the biophysical properties of the WT receptor are very similar between the two systems (44). The lipid composition of oocytes appear to be quite similar to that of a neuronal membrane, although the defined lipid profile in both sets of membranes does vary depending on the methods used for quantifying the different lipids (45, 46, 47, 48). On the other hand, the lipid composition of cultured HEK293T cells clearly lacks polyunsaturated fatty acids (49). Polyunsaturated fatty acids make up between 40 and 50% of fatty acids in neuronal membranes but less than 20% in cultured HEK293T cells (50, 51). This change in lipid composition is likely to have a dominant effect on both the fluidity of the bilayer and the formation of lipid nanodomains. Given that lipid composition has a dramatic effect on the coupling of binding and gating in the *Torpedo* muscle–like nAChR function, it may be that the effects of mutations studied here are more dramatic when the nAChR is imbedded in an unfavorable membrane environment.

## Experimental procedures

### Molecular biology and electrophysiology

Mutants were created from WT human α1, β1, δ, and ε nAChR sequences in the pcDNA3 vector using QuikChange Site-Directed Mutagenesis kits (Agilent) and verified by sequencing (35). The resulting vectors were linearized and capped circular RNA (cRNA) produced by *in vitro* transcription using the mMESSAGE mMACHINE T7 kit (Ambion).

Stage V–VI oocytes were injected with 5 ng of mutated α1 subunit cRNA along with 2.5 ng each of WT β1, δ, and ε subunit cRNA, and allowed to incubate 1 to 4 days at 16 ˚C in ND96^+^ buffer (96 mM NaCl, 2 mM KCl, 1 mM MgCl_2_, 1 mM CaCl_2_ 50 mM Hepes, 2 mM pyruvate, 10 ml/l penicillin/streptomycin, 50 mg/ml kanamycin, pH = 7.5). Whole-cell currents were measured in response to ACh concentration jumps using a TEVC apparatus (OC-725C oocyte clamp) in the presence of 1 μM atropine to prevent activation of endogenous calcium–activated chloride channels *via* muscarinic ACh receptors. Whole-cell currents were recorded in Hepes buffer (96 mM NaCl, 2 mM KCl, 1.8 mM BaCl_2_, 1 mM MgCl_2_, and 10 mM Hepes, pH 7.3), with the transmembrane voltage clamped at voltages between −20 mV and −80 mV, depending on the levels of protein expression. Dose responses for each mutant were acquired from at least two different batches of oocytes. Each individual dose response was fit with a variable slope sigmoidal dose–response curve. Plots were created using GraphPad Prism (GraphPad Software, Inc), and the individual pEC_50_ (−logEC_50_) values and Hill coefficients from each experiment averaged to give the presented values ± standard deviation. For the presented dose–response curves, the individual dose responses were normalized, and then each data point averaged. Curve fits of the averaged data are presented, with the error bars representing the standard error. Statistical significance was tested using a one-way ANOVA, followed by Dunnet’s post hoc test.

### Cell culture

HEK293T cells were maintained in a humidified atmosphere at 37 °C with 5% CO_2_, in Dulbecco’s modified Eagle’s medium supplemented with 5% heat-inactivated fetal bovine serum, 5% bovine calf serum, and 1% antibiotic–antimycotic (Gibco). Cells were plated in either 6-well dishes for the membrane potential assay or 12 cm dishes for the radioligand-binding assay at a density of 1.2 million cells/well. Transient transfection using polyethylenimine proceeded with a 2:1:1:1 ratio of nAChR subunits, α1:β1:δ:ε, adding up to a total of 2 μg of DNA for the membrane potential assay or 20 μg for the radioligand-binding assay. After 24 h, the cells were washed with 1× PBS at pH 7.4 and detached using 0.05% trypsin–EDTA, before they were resuspended in Dulbecco’s modified Eagle’s medium containing 1% fetal bovine serum/bovine calf serum and 1% antibiotic–antimycotic. Cells destined for the membrane potential assay were then seeded in a black-walled, clear-base, poly-d-lysine–coated, 384-well plate at a density of 45,000 cells/well. Cells destined for the radioligand-binding assay were transferred in 15 ml Falcon tubes, centrifuged at 1000 rpm for 2 min, and resuspended in 3.5 ml of phosphate ringer buffer (PRB; 140 mM KCl, 5.4 mM NaCl, 1.8 mM CaCl_2_, 1.7 mM MgCl_2_, 25 mM Hepes, 30 mg/l bovine serum albumin, pH = 7.4).

### Membrane potential assay

Changes in membrane potential in HEK293T cells transfected with WT and mutant nAChRs were measured using the FLIPR Tetra system (Molecular Devices). A voltage-sensitive dye, DiSBAC1(3) (FIVEphoton Biochemicals), was prepared by dissolving the powder in dimethyl sulfoxide. An assay buffer containing 2.5 μM DiSBAC1(3), 200 μM Direct Blue 71 (Sigma–Aldrich), and 1× Hanks’ balanced salt solution, 20 mM Hepes, pH 7.4 was freshly prepared as well. Cell medium was removed from the 384-well plate and replaced with 20 μl of the assay buffer. Cells were then incubated with the assay buffer at 37 °C for 30 min before using the FLIPR Tetra system to run the experiment. Prior to any additions, baseline fluorescence levels (λ_excitation_ = 510–545 nm, λ_emission_ = 565–625 nm) were measured every 2 s for 20 s. At 20 s, 10 μl of each ACh concentration was added onto each well, and the emitted fluorescence was monitored every 2 s for a total of 1000 s. In each experiment, four wells for each concentration were averaged to yield the presented curves in Fig. S2. The change in fluorescence for each ACh concentration was taken as the difference in fluorescence at 1000 s and the fluorescence prior to ACh addition. The change in fluorescence at each ACh concentration was then normalized to the maximum change in fluorescence and fit with a variable slope sigmoidal dose–response curve. Plots were created using GraphPad Prism, and the individual pEC_50_ (−logEC_50_) values and Hill coefficients from each experiment averaged to give the presented values ± standard deviation.

### Radioligand-binding assays

Cell surface in HEK293T cells was determined using the high-affinity radiolabeled toxin, [^125^I]-α-BTX. About 450 μl of HEK293T cells suspended in PRB were transferred into 2 ml Eppendorf tubes for each replicate of each mutant in the experiment. These cells were then rotated for 1 h at room temperature with a final concentration of 25 μM α-BTX (1:100 ratio of radiolabeled to nonradiolabeled toxin). Following incubation, cells were pelleted and excess α-BTX removed before the cells resuspended in toxin-free PRB. Using a filtration manifold, each sample was filtered through glass GF/C filters (Whatman) for 5 s, followed by 3 × 2 ml washes with PRB. Filters were then allowed to dry under suction for an additional 15 s to remove excess buffer. Bound [^125^I]-α-BTX was then quantified by γ counting each filter paper, and nonspecific binding was determined using the same procedure with untransfected cells.

### Homology models

Homology models of each human adult muscle nAChR subunit were created based on the 2.7 Å resolution structure of the muscle nAChR from *Torpedo* (Protein Data Bank: 6UWZ) (14) using the Swiss-Model online server (https://swissmodel.expasy.org/).

## Data availability

All data described here are available within the article and supporting information.

## Supporting information

This article contains supporting information.

## Conflict of interest

The authors declare that they have no conflicts of interest with the contents of this article.

## Supporting information

Supplemental Tables S1–S4

Supplemental Figure S1 **Functional effects of every M4 alanine mutant on nAChR function. Changes in EC**_**50**_**relative to WT for M4 alanine mutant are heat mapped onto each subunit.** Residues colored red correspond to gain-of-function mutants, those colored blue loss-of-function mutants, and those colored white cause no change in EC_50_

Supplemental Figure S2 **Effect of αM4 mutations on the function and expression of the nAChR in HEK293T cells.***A*, exemplary fluorescence traces for WT and untrasfected cells (*top*), αM4 mutants that were functionally expressed (*middle*), and nonexpressing/nonfunctional αM4 mutants (*bottom*) from the membrane potential assay. Each *colored line* corresponds to a different ACh concentration (0, *black*; 1 nM, *red*; 10 nM, *blue*; 100 nM, *green*; 1 μM, *pink*; 10 μM, *gold*; 100 μM, *navy*; 1 mM, *burgundy*). *B*, for mutants that responded to agonist, changes in fluorescence for each ACh concentration were normalized and plotted as dose-response curves. *C*, normalized surface expression for each mutant that did not respond to agonist are compared to WT and untransfected cells. ∗Denotes mutants that expressed significantly less than WT but significantly more than untransfected controls
