# Supplementary material for: Distinct functional roles for the M4 α-helix from each homologous subunit in the heteropentameric ligand-gated ion channel nAChR
Source: J Biol Chem. 2022 Jun 7;298(7):102104. doi: 10.1016/j.jbc.2022.102104 (PMC9260303; doi:10.1016/j.jbc.2022.102104)
Supplement: Supplemental Tables S1–S4 [file mmc1.docx]

**TABLE S1**

**Effects of Ala mutations in αM4 on nAChR function**

Dose response^a^

Mutant EC_50_ (μM) pEC_50_ (M) Hill slope n

WT 7.61 5.12 ± 0.07 1.70 ± 0.47 50

G437A 6.12 5.22 ± 0.06 1.58 ± 0.43 9

Q436A 8.29 5.08 ± 0.04 1.94 ± 0.14 13

Q435A 8.07 5.09 ± 0.03 2.03 ± 0.12 13

N434A 8.09 5.09 ± 0.04 1.94 ± 0.12 11

L433A 10.3 4.99 ± 0.04^b^ 1.93 ± 0.14 12

E432A 8.83 5.05 ± 0.03 1.92 ± 0.13 11

I431A 8.70 5.06 ± 0.03 1.85 ± 0.19 10

L430A 8.63 5.07 ± 0.07 1.72 ± 0.17 10

R429A 40.0 4.41 ± 0.11^b^ 1.09 ± 0.12 10

G428A 6.15 5.21 ± 0.04 1.66 ± 0.28 9

F426A 2.02 5.71 ± 0.14^b^ 1.80 ± 0.76 11

V425A 4.30 5.37 ± 0.05^b^ 1.89 ± 0.28 10

L423A 5.80 5.24 ± 0.07^b^ 2.54 ± 0.66 10

T422A 31.2 4.52 ± 0.13^b^ 1.40 ± 0.12 10

G421A 10.0 5.00 ± 0.05^b^ 1.96 ± 0.26 9

I420A 5.97 5.23 ± 0.07^b^ 2.71 ± 0.33 10

I419A 6.58 5.19 ± 0.07 2.15 ± 0.32 10

C418A 10.6 4.99 ± 0.13^b^ 1.82 ± 0.33 9

V417A 7.71 5.12 ± 0.08 1.94 ± 0.11 10

L416A 7.11 5.15 ± 0.05 2.01 ± 0.09 10

M415A 12.5 4.92 ± 0.13^b^ 1.61 ± 0.27 10

F414A 4.47 5.36 ± 0.09^b^ 1.76 ± 0.34 10

V413A 7.24 5.14 ± 0.07 2.20 ± 0.26 10

G412A 6.77 5.17 ± 0.07 1.54 ± 0.31 9

L411A 9.86 5.02 ± 0.10^b^ 2.35 ± 0.40 13

L410A 5.21 5.27 ± 0.06^b^ 2.06 ± 0.59 21

I409A 8.22 5.10 ± 0.10 2.05 ± 0.51 10

H408A 10.1 5.00 ± 0.05^b^ 1.80 ± 0.27 10

D407A 4.97 5.31 ± 0.04^b^ 3.12 ± 0.78 11

M406A 8.37 5.08 ± 0.04 1.70 ± 0.21 14

V405A 7.76 5.11 ± 0.06 2.03 ± 0.39 10

M404A 8.05 5.10 ± 0.04 2.14 ± 0.40 10

V402A 7.91 5.10 ± 0.02 2.05 ± 0.13 9

Y401A 9.86 5.01 ± 0.03^b^ 1.75 ± 0.48 11

K400A 12.9 4.90 ± 0.09^b^ 1.89 ± 0.63 15

W399A 8.90 5.05 ± 0.03 2.51 ± 0.41 10

^a^Measurements performed 1-4 days after cRNA injection (V_hold_ ranging from -20 to -80 mV). Error values represented as standard deviation

^b^p < 0.001 relative to WT via one-way ANOVA followed by Dunnet’s post hoc test

^c^No significant current observed up to 4 day after cRNA injection

**TABLE S2**

**Effects of Ala mutations in βM4 on nAChR function**

Dose response^a^

Mutant EC_50_ (μM) pEC_50_ (M) Hill slope n

WT 7.61 5.12 ± 0.07 1.70 ± 0.47 50

P478A 5.29 5.29 ± 0.11 1.59 ± 0.20 10

F477A 6.27 5.21 ± 0.10 1.59 ± 0.13 8

P476A 4.98 5.33 ± 0.20^b^ 1.49 ± 0.34 13

D475A 4.15 5.39 ± 0.11^b^ 1.27 ± 0.31 8

P474A 6.20 5.22 ± 0.13 1.27 ± 0.24 12

P273A 8.04 5.11 ± 0.11 1.29 ± 0.29 11

P472A 7.91 5.11 ± 0.10 1.21 ± 0.20 9

L471A 7.44 5.14 ± 0.10 1.63 ± 0.71 11

H470A 13.0 4.90 ± 0.11^b^ 1.24 ± 0.19 13

Y469A 7.76 5.12 ± 0.10 1.20 ± 0.10 9

T468A 7.54 5.13 ± 0.07 1.34 ± 0.15 11

D466A 5.08 5.31 ± 0.14^b^ 1.57 ± 0.48 11

L465A 9.71 5.03 ± 0.11 1.20 ± 0.22 11

F464A 8.55 5.08 ± 0.11 1.38 ± 0.19 10

I463A 3.67 5.44 ± 0.16^b^ 1.78 ± 0.11 8

V462A 7.79 5.12 ± 0.10 1.57 ± 0.79 11

L461A 6.78 5.17 ± 0.05 1.57 ± 0.38 9

T460A 8.84 5.06 ± 0.09 1.56 ± 0.15 11

G459A 14.4 4.90 ± 0.28^b^ 1.12 ± 0.30 10

V458A 7.03 5.16 ± 0.11 1.73 ± 0.45 8

S457A 13.2 4.93 ± 0.22^b^ 1.10 ± 0.24 11

T456A 8.26 5.12 ± 0.18 1.24 ± 0.18 8

F455A 6.72 5.18 ± 0.09 1.73 ± 0.66 10

I454A 6.07 5.25 ± 0.20 1.46 ± 0.45 11

I453A 4.57 5.36 ± 0.12^b^ 1.46 ± 0.24 8

F452A 10.5 4.98 ± 0.07 1.69 ± 0.11 8

T451A 10.4 5.00 ± 0.11 1.25 ± 0.24 11

W450A 12.7 4.94 ± 0.19^b^ 1.27 ± 0.20 14

L449A 15.9 4.82 ± 0.15^b^ 1.45 ± 0.19 8

F448A 4.36 5.38 ± 0.15^b^ 1.63 ± 0.48 8

L447A 9.14 5.06 ± 0.16 1.24 ± 0.23 8

R446A 12.6 4.92 ± 0.14 1.18 ± 0.23 9

D445A 11.9 4.99 ± 0.30 1.46 ± 0.13 9

V444A 2.22 5.66 ± 0.07^b^ 1.89 ± 0.30 11

V443A 9.98 5.02 ± 0.14 1.16 ± 0.21 11

M442A 11.3 4.96 ± 0.13 1.45 ± 0.29 12

V440A 7.28 5.14 ± 0.08 1.49 ± 0.30 12

F439A 7.11 5.15 ± 0.07 1.80 ± 0.08 9

Q438A 7.92 5.10 ± 0.02 1.33 ± 0.22 9

W437A 8.88 5.07 ± 0.12 1.56 ± 0.26 9

^a^Measurements performed 1-4 days after cRNA injection (V_hold_ ranging from -20 to -80 mV). Error values represented as standard deviation

^b^p < 0.001 relative to WT via one-way ANOVA followed by Dunnet’s post hoc test

^c^No significant current observed up to 4 day after cRNA injection

**TABLE S3**

**Effects of Ala mutations in δM4 on nAChR function**

Dose response^a^

Mutant EC_50_ (μM) pEC_50_ (M) Hill slope n

WT 7.61 5.12 ± 0.07 1.70 ± 0.47 50

Q479A 5.96 5.24 ± 0.12 1.70 ± 0.11 8

P478A 4.72 5.34 ± 0.11^b^ 1.43 ± 0.56 7

P477A 11.5 4.94 ± 0.09^b^ 1.26 ± 0.14 8

P476A 8.28 5.08 ± 0.04 1.63 ± 0.15 8

Q475A 6.60 5.19 ± 0.10 1.14 ± 0.32 8

N474A 7.50 5.13 ± 0.09 1.74 ± 0.17 8

Y473A 6.84 5.17 ± 0.09 1.49 ± 0.26 8

V472A 8.78 5.08 ± 0.15 1.22 ± 0.26 7

G471A 14.1 4.86 ± 0.11^b^ 1.57 ± 0.25 4

Q470A 7.36 5.15 ± 0.14 1.43 ± 0.29 7

L469A 5.13 5.30 ± 0.09^b^ 1.71 ± 0.38 9

F468A 5.83 5.25 ± 0.10 1.60 ± 0.27 7

I467A 4.63 5.34 ± 0.07^b^ 1.91 ± 0.79 7

W466A 5.49 5.28 ± 0.14^b^ 1.57 ± 0.27 8

T464A 8.24 5.09 ± 0.08 1.28 ± 0.14 7

G463A 4.88 5.32 ± 0.10^b^ 1.71 ± 0.38 8

V462A 7.41 5.15 ± 0.12 1.45 ± 0.27 8

V461A 7.13 5.15 ± 0.07 1.60 ± 0.27 9

M460A 7.23 5.15 ± 0.07 1.52 ± 0.22 8

V459A 10.2 5.00 ± 0.12 1.31 ± 0.19 8

P458A 4.08 5.40 ± 0.09^b^ 2.06 ± 0.75 7

T457A 7.84 5.12 ± 0.13 1.38 ± 0.15 7

V456A 5.06 5.33 ± 0.19^b^ 1.58 ± 0.41 8

V455A 5.77 5.26 ± 0.16 1.78 ± 0.50 8

F454A 7.67 5.12 ± 0.05 1.64 ± 0.17 8

L453A 7.66 5.12 ± 0.08 1.39 ± 0.24 8

C452A 4.86 5.32 ± 0.07^b^ 1.56 ± 0.16 11

L451A 7.46 5.14 ± 0.11 1.60 ± 0.22 9

R450A 5.15 5.29 ± 0.04^b^ 1.72 ± 0.17 7

D449A 5.03 5.34 ± 0.20^b^ 2.00 ± 0.19 8

V448A 8.53 5.07 ± 0.05 1.63 ± 0.07 8

T447A 6.57 5.19 ± 0.07 1.85 ± 0.15 8

R446A 7.87 5.11 ± 0.09 1.54 ± 0.17 8

V444A 4.35 5.36 ± 0.03^b^ 2.02 ± 0.17 8

R443A 9.66 5.03 ± 0.13 1.34 ± 0.22 7

N442A 7.82 5.11 ± 0.08 1.34 ± 0.18 7

W441A 5.71 5.24 ± 0.01 1.80 ± 0.07 3

^a^Measurements performed 1-4 days after cRNA injection (V_hold_ ranging from -20 to -80 mV). Error values represented as standard deviation

^b^p < 0.001 relative to WT via one-way ANOVA followed by Dunnet’s post hoc test

^c^No significant current observed up to 4 day after cRNA injection

**TABLE S4**

**Effects of Ala mutations in εM4 on nAChR function**

Dose response^a^

Mutant EC_50_ (μM) pEC_50_ (M) Hill slope n

WT 7.61 5.12 ± 0.07 1.70 ± 0.47 50

P473A 8.76 5.07 ± 0.09 1.79 ± 0.11 8

Q472A 6.02 5.22 ± 0.04 1.71 ± 0.13 8

I471A 15.5 4.81 ± 0.07^b^ 1.61 ± 0.24 10

C470A 13.4 4.88 ± 0.05^b^ 1.54 ± 0.08 12

P469A 6.68 5.18 ± 0.03 1.92 ± 0.36 10

Y467A 7.51 5.14 ± 0.10 1.58 ± 0.16 8

P466A 7.90 5.11 ± 0.09 1.54 ± 0.08 8

L465A 7.72 5.12 ± 0.07 1.73 ± 0.24 9

D464A 8.06 5.10 ± 0.06 1.96 ± 0.59 8

P463A 12.1 4.93 ± 0.08^b^ 1.77 ± 0.10 9

V462A 6.88 5.17 ± 0.06 1.52 ± 0.15 8

R461A 7.42 5.13 ± 0.05 1.73 ± 0.07 8

N460A 7.63 5.13 ± 0.11 1.66 ± 0.13 8

F459A 6.35 5.20 ± 0.07 1.63 ± 0.11 8

Y458A 5.10 5.30 ± 0.07^b^ 1.43 ± 0.20 8

G456A 7.95 5.11 ± 0.09 1.51 ± 0.07 8

L455A 7.46 5.13 ± 0.04 1.72 ± 0.12 8

F454A 3.90 5.42 ± 0.10^b^ 1.80 ± 0.25 8

I453A 4.50 5.35 ± 0.06^b^ 1.83 ± 0.27 8

L452A 7.96 5.10 ± 0.05 1.64 ± 0.12 8

S451A 8.70 5.06 ± 0.06 1.77 ± 0.10 9

S450A 8.05 5.09 ± 0.02 1.89 ± 0.25 8

G449A 5.62 5.26 ± 0.12^b^ 1.54 ± 0.52 9

V448A 7.52 5.13 ± 0.05 1.69 ± 0.15 8

S447A 9.33 5.03 ± 0.05 1.66 ± 0.18 8

F446A 8.89 5.06 ± 0.07 1.51 ± 0.18 9

L445A 8.04 5.11 ± 0.10 1.53 ± 0.28 8

V444A 7.40 5.14 ± 0.08 1.48 ± 0.13 8

L443A 8.08 5.10 ± 0.08 1.69 ± 0.13 11

W440A 7.48 5.13 ± 0.04 1.57 ± 0.15 8

F439A 8.59 5.07 ± 0.07 1.81 ± 0.08 8

C438A 10.1 5.00 ± 0.06^b^ 1.67 ± 0.20 9

I437A 7.58 5.13 ± 0.08 1.83 ± 0.22 8

N436A 12.9 4.90 ± 0.09^b^ 1.76 ± 0.22 11

D435A 8.03 5.10 ± 0.05 1.73 ± 0.18 8

L434A 8.07 5.10 ± 0.08 1.89 ± 0.21 8

N432A 8.66 5.07 ± 0.06 1.69 ± 0.11 8

G431A 10.6 4.99 ± 0.11^b^ 1.60 ± 0.17 8

M430A No current^c^ 8

R429A 7.29 5.14 ± 0.04 1.54 ± 0.21 8

V428A 5.63 5.25 ± 0.06^b^ 1.56 ± 0.19 8

W427A 5.85 5.25 ± 0.14^b^ 1.37 ± 0.39 9

Same legend as previous three tables
