# Supplementary figures and images for: Distinct functional roles for the M4 α-helix from each homologous subunit in the heteropentameric ligand-gated ion channel nAChR
Source: J Biol Chem. 2022 Jun 7;298(7):102104. doi: 10.1016/j.jbc.2022.102104 (PMC9260303; doi:10.1016/j.jbc.2022.102104)

Figure S1

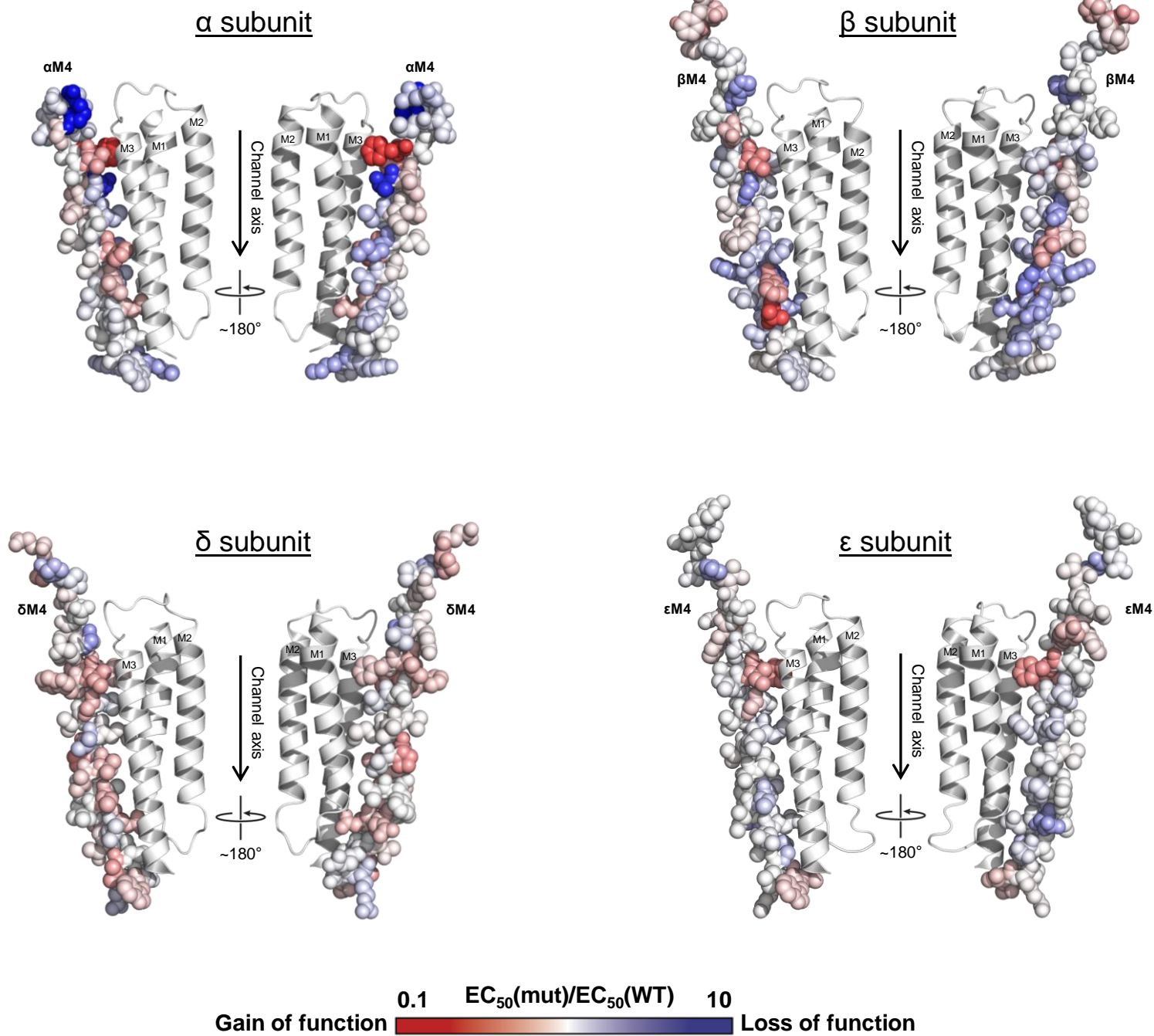

Supplement: Supplemental Figure S1 — Functional effects of every M4 alanine mutant on nAChR function. Changes in EC50relative to WT for M4 alanine mutant are heat mapped onto each subunit. Residues colored red correspond to gain-of-function mutants, those colored blue loss-of-function mutants, and those colored white cause no change in EC50 [file mmc2.pdf]

A

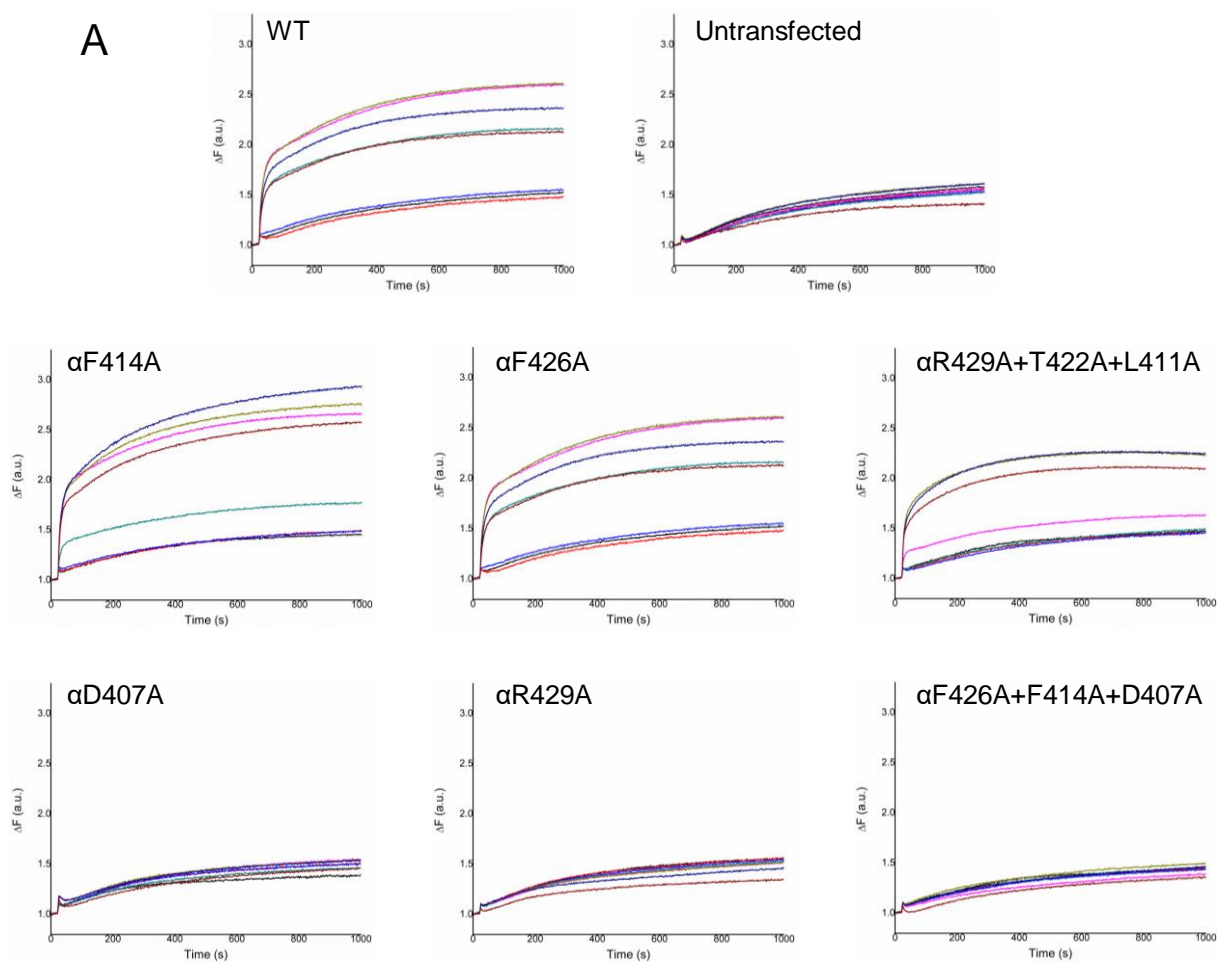

B

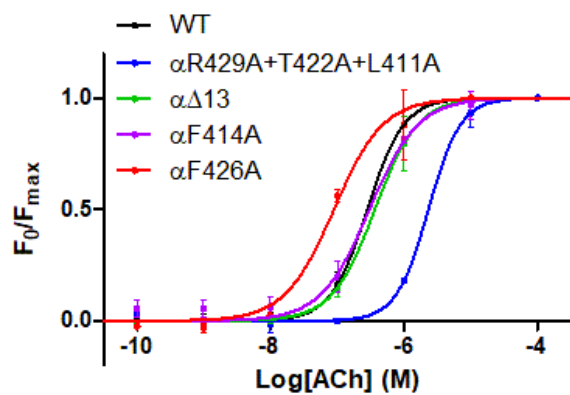

C

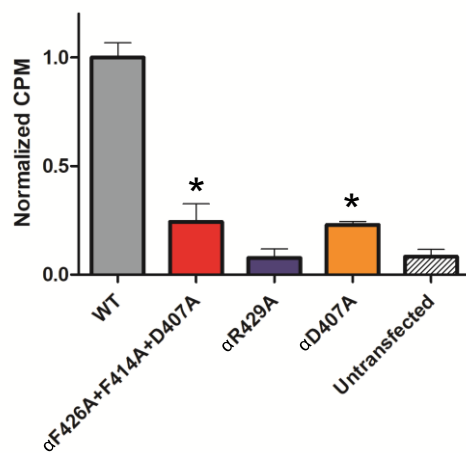

Supplement: Supplemental Figure S2 — Effect of αM4 mutations on the function and expression of the nAChR in HEK293T cells.A, exemplary fluorescence traces for WT and untrasfected cells (top), αM4 mutants that were functionally expressed (middle), and nonexpressing/nonfunctional αM4 mutants (bottom) from the membrane potential assay. Each colored line corresponds to a different ACh concentration (0, black; 1 nM, red; 10 nM, blue; 100 nM, green; 1 μM, pink; 10 μM, gold; 100 μM, navy; 1 mM, burgundy). B, for mutants that responded to agonist, changes in fluorescence for each ACh concentration were normalized and plotted as dose-response curves. C, normalized surface expression for each mutant that did not respond to agonist are compared to WT and untransfected cells. ∗Denotes mutants that expressed significantly less than WT but significantly more than untransfected controls [file mmc3.pdf]
